# Gut microbiota-derived short-chain fatty acids (SCFAs): immunomodulatory effects and therapeutic potential in infections

**DOI:** 10.1128/cmr.00368-25

**Published:** 2026-06-10

**Authors:** Junxiu Yu, Wenweiran Li, Yajie Xu, Jianguo Tang

**Affiliations:** 1 Department of Trauma-Emergency & Critical Care Medicine, Shanghai Fifth People’s Hospital, Fudan University, Shanghai, China; University of Minnesota Medical School Twin Cities, Minneapolis, Minnesota, USA

**Keywords:** short-chain fatty acids, gut microbiota, anti-infective, microbiota-host interaction, intestinal barrier, adjuvant therapy

## Abstract

The human gut microbiotas constitute a complex microecosystem essential for host homeostasis. Among its metabolites, short-chain fatty acids (SCFAs) act as key signaling molecules linking microbial activity to host immunity and barrier function. Based on existing literature, we conducted a comprehensive analysis of the interaction between SCFAs and 11 key gut pathogens. These pathogens include bacteria (i.e., *Staphylococcus aureus*, *Clostridioides difficile, Salmonella* spp.*, Campylobacter jejuni, Klebsiella pneumoniae, Vibrio cholerae*), fungi (i.e., *Candida albicans*), and viruses (i.e., SARS-CoV-2, influenza virus, respiratory syncytial virus, rotavirus). In terms of antibacterial effects, SCFAs exhibit antibacterial activity against most gut microorganisms, but they support the colonization of a few species (i.e., *Campylobacter jejuni*). Given the complex interactions between SCFAs and the gut microbiota, as well as their regulatory roles in infection, further investigation of the microbiota-SCFA axis is essential for developing effective strategies to prevent and treat infectious diseases. This review systematically summarizes the mechanism and clinical evidence of SCFAs in microbial infections to provide novel ideas for infectious disease management.

## INTRODUCTION

**Fig 1:**
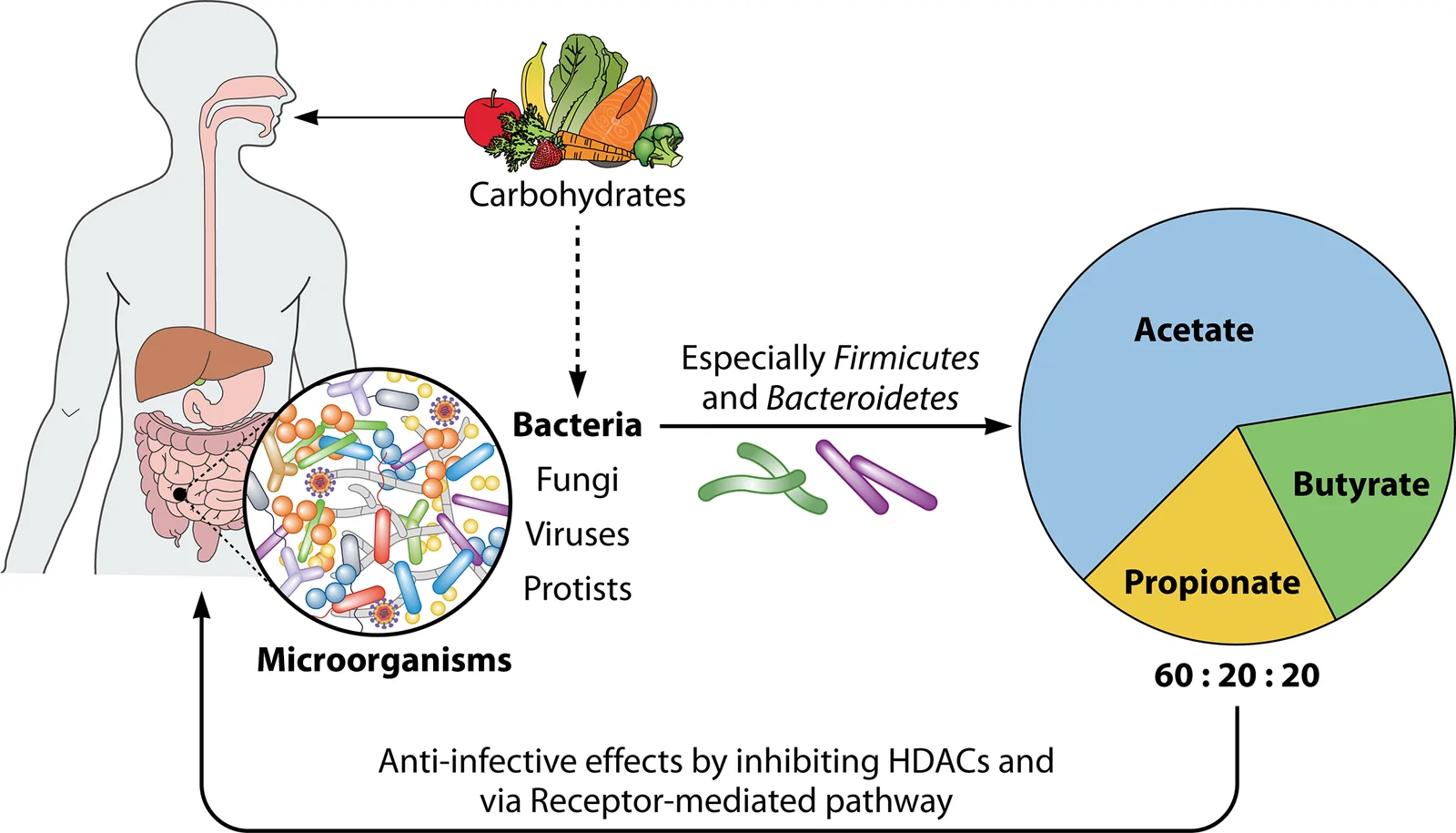
Dietary carbohydrates, fermented by intestinal bacteria, produce acetate, propionate, and butyrate in a 60:20:20 ratio, exerting anti-infective effects by inhibiting HDACs and via receptor-mediated pathway.

The human gastrointestinal tract harbors more than 100 trillion microorganisms, collectively referred to as the gut microbiota or microbiome, including bacteria, fungi, viruses, and helminths (1). Short-chain fatty acids (SCFAs) refer to fatty acids containing fewer than six carbon atoms. They are produced by the fermentation of carbohydrates by bacteria from the phyla Bacteroidetes and Firmicutes (2), with an approximate ratio of acetate to propionate to butyrate of 60:20:20. Due to their high water solubility, SCFAs are readily absorbed by the body (1). SCFAs exert anti-infective effects mainly by inhibiting histone deacetylases (HDACs) and mediating through G protein-coupled receptors (GPRs). Therefore, studying the gut microbiota-SCFA axis plays a key role in anti-infection. This review focuses on SCFAs, systematically organizing their regulatory relationship with gut microbiota, providing an in-depth analysis of the mechanisms and clinical evidence of SCFAs against gut microbial infections. It aims to provide a new perspective for understanding the link between gut microecology and infection, and to offer a theoretical basis and practical directions for the prevention and treatment of infectious diseases (Fig. 1).

## METABOLISM AND TRANSPORT OF SCFAs

SCFAs show a stepped concentration gradient in the colon, with total concentrations decreasing from 70–140 mM in the proximal colon to 20–70 mM in the distal colon (3). The daily production of SCFAs is about 400–600 mmol, which can provide about 10% of the body’s energy needs (4). Carbohydrates are the main source for SCFA production. In addition, proteins (except branched-chain amino acids) and peptides can serve as precursors to produce minor amounts of SCFAs (5, 6). SCFAs reduce intestinal lumen pH by exchanging with bicarbonate, inhibiting the growth of many pathogenic bacteria (3). The pH value gradually increases from the cecum to the rectum (about 5.5 in the proximal colon and 6.5 in the distal colon [7]), a low-pH environment that favors the growth of butyrate-producing bacteria and suppresses the excessive proliferation of pH-sensitive pathogens like Enterobacteriaceae (8–10).

Different types of SCFAs have different production pathways. Acetate mainly originates from Bacteroidetes, synthesized via two core pathways—pyruvate decarboxylation and CO_2_ reduction (11, 12). Propionate is produced by specific microbiota through three pathways (succinate, acrylate, 1,2-propanediol), with some Bacteroidetes involved in key steps (13). Butyrate is produced by obligate anaerobic Clostridium clusters (I, III, IV, XI, XIVa, XV, XVI), including *Faecalibacterium prausnitzii* and *Roseburia intestinalis*, whose synthesis requires catalysis by butyryl-CoA and acetyl-CoA transferase (14).

SCFAs are utilized through two metabolic pathways: first, SCFAs are absorbed mainly in the intestine (primarily the colon). The absorption process mainly depends on the sodium-coupled monocarboxylate transporter (SMCT1, Na^+^-dependent) and the pH-dependent hydrogen-coupled monocarboxylate transporter (MCT1/4, H^+^-dependent) for active transport into intestinal epithelial cells (15). Only a small amount of non-ionized SCFAs is absorbed through passive diffusion (1).

**Fig 2:**
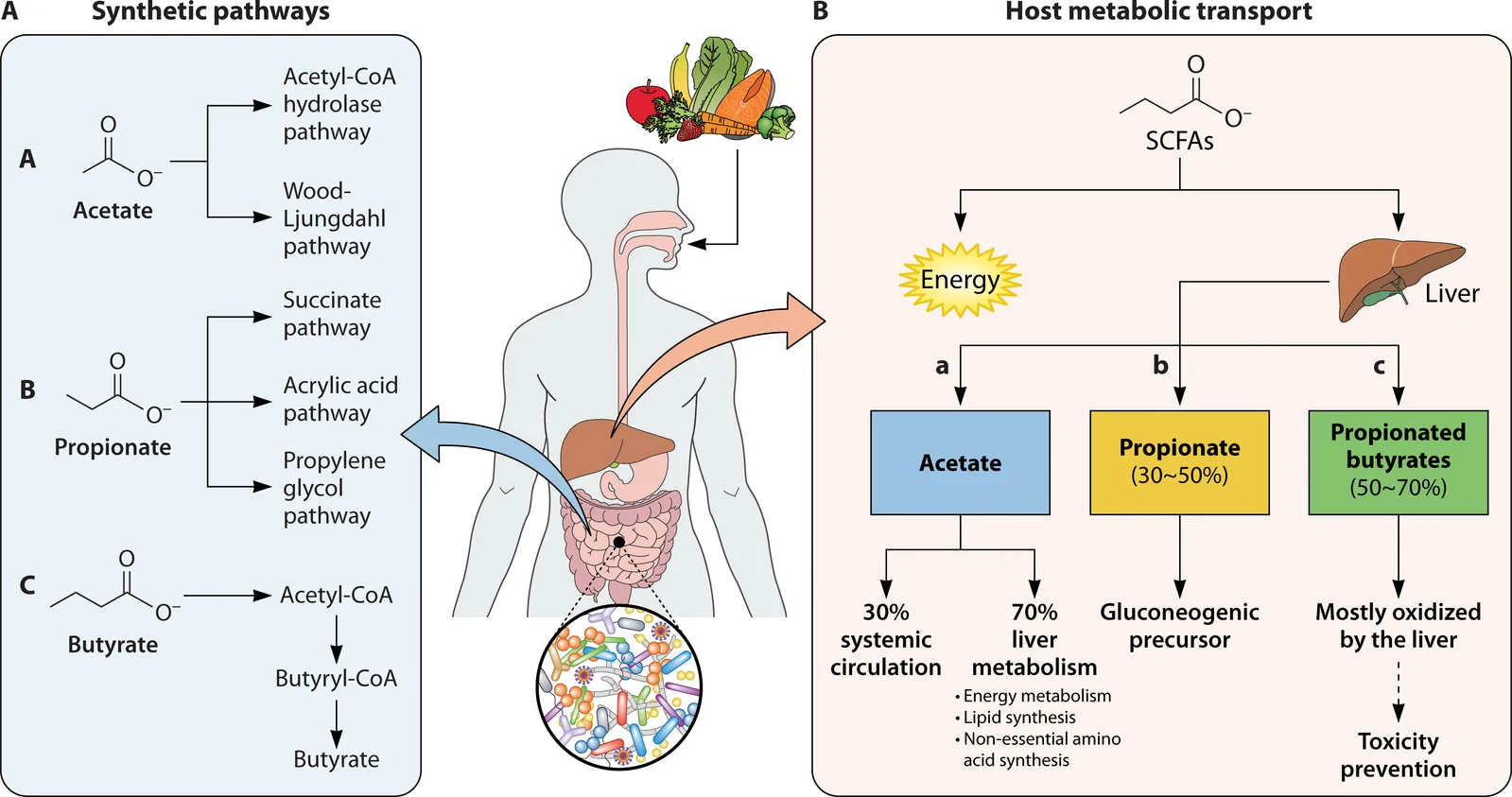
Synthesis pathways of SCFAs (panel **A**) and host metabolic transport (panel **B**). SCFA synthesis pathways by gut microbiota (panel **A**): (A) acetate synthesis: mainly via the “acetyl-CoA hydrolysis pathway” and the “Wood-Ljungdahl specific pathway”; (B) propionate synthesis: synthesized through the “succinate pathway,” “acrylate pathway,” and “propanediol pathway”; (C) butyrate synthesis: acetyl-CoA undergoes condensation-reduction reactions to form butyryl-CoA, which is then converted to butyrate via two terminal pathways. SCFA metabolism and transport (panel **B**): most SCFAs are directly oxidized in colonocytes to obtain energy. SCFAs not utilized by colonocytes are transported to the liver via the portal vein. Once SCFAs enter the liver: (a) 30% of acetate enters systemic circulation, while 70% is metabolized in the liver, participating in energy metabolism, lipid synthesis, amino acid metabolism, etc. (b) 30%–50% of propionate serves as a precursor for hepatic gluconeogenesis. (c) 50%–70% of propionate and butyrate are mostly oxidized by the liver to prevent their accumulation in the blood and potential toxicity.

Second, post-absorption metabolism has two routes: (i) metabolism in colon cells: SCFAs are directly oxidized to produce energy, accounting for 60%–70% of colon cell energy supply (3). Butyrate is the preferred energy substrate (oxidized to ketone bodies and CO_₂_), while acetate also plays an important role in energy supply (3). (ii) Transport to the liver via the portal vein: unused SCFAs enter the liver through the basolateral membrane. Approximately 70% of acetate is taken up by the liver for metabolic processes, with the rest entering the systemic circulation (16, 17). Propionate serves as a gluconeogenic precursor in hepatocytes (30%–50% liver uptake rate) (18). However, most propionate and butyrate are cleared by the liver to avoid blood accumulation and toxicity, resulting in only trace amounts reaching the systemic circulation (Fig. 2).

## CHEMICAL DIVERSITY OF SCFAs

### Carbon chain length

Differences in the carbon chain length of SCFAs affect their key biological effects, mainly reflected in the following aspects.

In terms of physical and chemical properties and *in vivo* distribution, the water solubility of SCFAs decreases with the extension of the carbon chain, while the fat solubility increases in the opposite direction (19, 20): C2 (acetate) has the strongest water solubility, distributes widely throughout the body, and can penetrate the blood-brain barrier (the concentration in cerebrospinal fluid is about 35 μM (21)). C3 (propionate) and C4 (butyrate) have moderate fat solubility, with both membrane permeability and intracellular action ability, and are the core SCFAs mediating local and systemic regulation. C5 (valerate) and C6 (caproate) have significantly enhanced fat solubility but extremely low water solubility, which limits their free concentration in the intestinal lumen and mainly acts locally in the colon.

In terms of receptor binding, SCFAs have strict carbon chain selectivity for the activation of GPRs: GPR43 (FFAR2) has the highest affinity for C2 and C3; GPR41 (FFAR3) prefers C3 and C4; GPR109a only recognizes SCFAs with a carbon chain ≥4, among which butyric acid has significantly higher activation efficiency than valeric acid; Olfactory receptor Olfr78 can bind to acetate and propionate, but not to butyrate (22), and exhibits the highest affinity for propionate (23).

In the process of metabolism and transport, short carbon chains (C2, C3) preferentially enter cells through the SMCT1, while medium and long carbon chains (C4–C6) mainly rely on MCT1/4 (24).

In addition to SCFAs, medium-chain fatty acids (MCFAs) also play important roles in intestinal barrier function, antibacterial defense, and immune homeostasis. MCFAs are saturated fatty acids containing 6–12 carbon atoms (25). Among them, caprylic acid (CA) exhibits stronger inhibitory effects on Gram-negative bacteria, such as *Escherichia coli* and *Salmonella*, than decanoic acid and lauric acid (26). CA is mainly obtained from natural diets including coconut oil, goat milk, and palm kernel oil (27). It can be rapidly absorbed in the intestine, transported via the portal vein, and quickly taken up and oxidized in the liver (28). In terms of intestinal barrier protection, CA strengthens the intestinal barrier by regulating gut microbiota structure and increasing SCFA levels (29). Meanwhile, it enhances the intestinal immune barrier and significantly reduces pathogen translocation across the epithelium by inhibiting HDACs activity and increasing histone acetylation of defensin genes (26). Regarding antibacterial effects, CA shows pH-dependent bactericidal activity. Under acidic conditions, it penetrates the bacterial cell membrane in a protonated hydrophobic form, dissociates intracellularly, disrupts membrane integrity, and blocks energy metabolism, thereby exerting direct bactericidal effects. Its bactericidal activity is significantly weakened under neutral conditions (27).

Notably, butyrate has a “butyrate paradox”: its effect on cells is not fixed but is regulated by cell metabolic status, intracellular concentration, and cell type (30). At physiological concentrations (14–28 mM in the colon, <2.5 mM in peripheral blood), it exerts anti-inflammatory and antiviral effects; at supraphysiological concentrations, it may inhibit type I interferon-stimulated gene expression to promote virus replication or trigger abnormal immune responses (30).

### Branched-chain short-chain fatty acids

Branched-chain fatty acids (BCFAs) are saturated fatty acids with one or more methyl branches in the carbon chain, mainly divided into iso-configuration (methyl branch at the penultimate position of the terminal), anteiso-configuration (methyl branch at the third position from the end of the terminal), and polymethyl type (31). A total of 4–6 carbon branched-chain SCFAs are a minor component of their family, accounting for 0.01% of the total BCFAs in dairy products, with extremely low absolute content in a single diet. Although they exist in small amounts in fermented foods and fish, their dietary contribution is negligible (31). BCFAs can indirectly participate in glucose and lipid metabolism by regulating intestinal flora and promoting intestinal hormone secretion, and at the same time, serve as specific markers of intestinal microbial protein fermentation to reflect intestinal health status (31, 32). In addition, studies have shown that the anticancer activity of iso-configured branched-chain fatty acids (e.g., iso-15:0 and iso-17:0) is significantly stronger than that of their anteiso-configured homologs (e.g., anteiso-15:0 and anteiso-17:0) (33). At present, there is a lack of special research on short-chain branched-chain fatty acids, and their specific dietary intake and metabolic molecular mechanisms are not clear, so targeted experiments are needed to further clarify their human health value.

### Short-chain fatty acid ester derivatives

SCFA ester derivatives are formed by the esterification reaction between the carboxyl group of SCFAs and alcohols or glycerol. Tributyrin (TB) is a typical representative and belongs to the prodrug of SCFAs (34). Its chemical structure directly determines receptor affinity, cellular uptake efficiency, and microbial metabolic pathways. Esterification modification blocks the carboxyl group of SCFAs, making the derivatives unable to directly bind to the corresponding receptors of free SCFAs (e.g., GPR41, GPR43) (35). The corresponding receptors can only be activated after free SCFAs are released by esterase hydrolysis. In addition, SCFA ester derivatives have improved lipophilicity, which significantly enhances membrane permeability and transmembrane transport efficiency. Their hydrolysis and transport rates increase with the increase of acyl carbon chain length, and the rates of straight-chain isomers are significantly higher than those of branched-chain isomers (36). For example, TB is a neutral lipophilic molecule that can directly cross cell membranes through passive diffusion, and its cellular uptake efficiency is much higher than that of free butyric acid (34). In terms of cellular metabolic pathways, studies have found that TB can significantly upregulate the expression of SCFA transporters MCT1/SMCT one in the cecum compared with sodium butyrate (NaB), thereby protecting the intestinal barrier and improving intestinal microbiota diversity (37). Furthermore, TB can effectively reverse antibiotic-induced intestinal microbiota disturbance, reduce intestinal inflammation and barrier damage, and the effect of low dose (0.3 g/kg) is significantly better than that of high dose (38).

## SIGNAL TRANSDUCTION OF SCFAs IN CELLS

SCFAs support the host to resist bacterial, fungal, and viral infections through two core signal transduction pathways: “receptor-mediated signal activation” and “epigenetic regulation.”

### Receptor-mediated anti-infective signal transduction of SCFAs

SCFAs transmit anti-infectious signals mainly through receptor-mediated pathways, and distinct receptors exert specific protective effects in particular infection contexts. The common receptors are GPR41, GPR43, GPR109a, and Olfr78 (39).

GPR43 and GPR41 are core receptors for SCFAs to mediate anti-infective signals. Although they belong to the same family, they have significant differences: GPR43 can bind to Gi/o proteins and Gq proteins to mediate its functions. Gi/o proteins inhibit adenylate cyclase and reduce intracellular cyclic adenosine monophosphate levels, whereas Gq proteins activate phospholipase Cβ and promote intracellular calcium ion release (40). It is mainly expressed in immune cells, the large intestine, and adipose tissue, and regulates inflammatory responses and glucose-lipid metabolism (35, 41–43). GPR41 only binds to Gi/o proteins and has a wider expression range, focusing on regulating energy balance (35, 41, 42).

GPR109a is distributed in the colonic epithelium, macrophages, adipose tissue, and pancreatic β cells, and butyrate is a potent agonist of this receptor (44, 45). In the intestine, it activates the NLRP3 inflammasome to promote IL-18 secretion, induces anti-inflammatory regulatory T cells (Tregs), and IL-10 producing CD4^+^ T cells (44), and acts as a nutrient sensor to enhance MCT-1 function (46). Outside the intestine, niacin activates it to inhibit macrophage inflammatory factor secretion and promote cholesterol efflux, resisting atherosclerosis (44).

Olfr78 is mainly expressed in neurons, enteroendocrine cells, colonic epithelial cells, and vascular smooth muscle (47). Propionate exerts anti-rectal cancer effects by binding to the Olfr78 receptor, increasing intracellular cAMP levels, and inhibiting the MEK/ERK signaling pathway (48).

### Anti-infective effects of SCFAs mediated by epigenetic regulation

SCFAs (especially butyrate and propionate) can regulate the expression of anti-infection-related genes by inhibiting HDACs and mediating short-chain lysine acylation.

SCFAs can enter cells and directly bind to HDACs to increase histone acetylation, opening chromatin structure to activate or inhibit anti-infection-related genes (49–52).

In addition to canonical histone acetylation, SCFAs also mediate a variety of short-chain lysine acylation reactions, such as crotonylation (Kcr) and propionylation (Kpr) (53). Butyrate can be converted into crotonyl-CoA, a precursor for histone crotonylation mediated by p300-CBP58, thereby promoting histone crotonylation (52). Through Kcr, the host can regulate the DNA damage response (e.g., DNA double-strand break repair) to maintain genome stability, enhance the transcription of anti-infective genes in immune cells, and strengthen the clearance of bacteria and viruses (54, 55). Meanwhile, butyrate and crotonic acid can inhibit class I histone deacetylases (HDAC 1-3) with decrotonylase activity, thus maintaining stable histone crotonylation levels (56). Propionate can be catalyzed by intracellular acyl-CoA synthetases to generate propionyl-CoA (57), and these coenzyme derivatives participate in anti-infective regulation by mediating acylation modifications. For instance, under physiological pH, propionyl-CoA can inhibit the activity of HilD, a virulence regulator of *Salmonella Typhimurium*, via propionylation modification, thereby suppressing the expression of invasion-related genes and the secretion of effector proteins in *Salmonella* pathogenicity island 1 (SPI1), and weakening the invasive capacity of the bacterium (58).

## MECHANISMS OF SCFAs IN RESISTING INFECTIONS

### SCFAs strengthen the intestinal epithelial barrier functions

SCFAs protect the intestinal barrier through four coordinated mechanisms. (i) Strengthening tight junctions: butyrate (1–5 mM) upregulates the expression and assembly of tight junction proteins, such as ZO-1, occludin, and claudin-1/3/4, by activating the adenosine monophosphate-activated protein kinase (AMPK) pathway and the signal transducer and activator of transcription 3/specificity protein 1 (STAT3/SP1) transcription factors (2, 59), and downregulates the permeability-promoting protein claudin-2 (promotes barrier leakage) through an IL-10 receptor-dependent pathway (60). Additionally, SCFAs show concentration dependence: physiological concentrations (1–10 mM) promote tight junction assembly, while high concentrations (≥50 mM) damage the structure (61–63). (ii) Maintaining mucus layer function: butyrate inhibits HDACs to upregulate the expression of mucin (MUC2), stimulating goblet cells to secrete MUC2. This enhances the resistance of the mucus layer to luminal pathogens, maintaining cell stability (64). (iii) Sustaining cell stability: SCFAs can inhibit the activation of NLRP3 inflammasome and promote autophagy, among which butyrate can also directly downregulate IL-1β levels to reduce the damage of inflammatory responses to the intestinal barrier (65). Meanwhile, butyrate can stabilize hypoxia-inducible factor (HIF-1α), a key transcription factor maintaining intestinal barrier integrity, to support colonocyte proliferation and repair and ensure the normal exertion of the barrier protective effect of SCFAs (66). In addition, butyrate can further maintain the stability of the intestinal barrier by inhibiting epithelial cell apoptosis and antagonizing oxidative stress damage (67). (iv) Promoting antimicrobial peptide (AMPs) secretion: butyrate relieves HDAC-mediated AMPs gene inhibition, activates the GPR109a-STAT3 pathway to enhance secretion (2), and propionate also can also promote AMPs secretion through the GPR43 receptor, jointly strengthening the mucosal immune barrier (68).

### SCFAs regulate host immune responses

#### T cell

High concentrations of SCFAs promote the differentiation of colonic Tregs or induce IL-10 secretion to alleviate colonic inflammation. In a GPR41- and GPR43-dependent manner, SCFAs enhance the memory potential, recall response capacity, and mitochondrial oxidative phosphorylation levels of activated CD8 +T cells (69). Acetate upregulates cortactin acetylation, disrupts the filamentous actin (F-actin) cytoskeleton in T cells, and thereby inhibits T cell migration, activation, and proliferation in a GPR43-independent manner (70). Propionate induces extrathymic Treg generation by HDACs activity. Butyrate also inhibits HDACs, increases histone H3 acetylation in the Foxp3 gene locus, promotes intestinalTreg differentiation, and upregulates IL-10 expression. Meanwhile, butyrate maintains the Th1/Th17 balance by upregulating T-bet and downregulating the Th17-associated transcription factor RORγt. Furthermore, butyrate enhances IFN-γ and granzyme B expression in CD8+ T cells via an HDACs inhibition pathway independent of GPR41/43 (71).

#### B cell

Low-concentration SCFAs promote intestinal IgA and systemic IgG production by upregulating activation-induced cytidine deaminase (AID) and B lymphocyte-induced maturation protein-1 (BLIMP-1) via HDAC inhibition. High-concentration SCFAs attenuate humoral immunity by upregulating microRNAs (miRNAs) targeting *Aicda* (encoding AID) and *Prdm1* (encoding BLIMP-1), and this regulatory effect is largely independent of GPR41/GPR43 (72). Among them, acetate promotes retinoic acid (RA) synthesis via GPR43 on dendritic cells (DCs), thereby upregulating IgA class-switch recombination and intestinal IgA secretion in B cells, and regulating the selective binding of IgA to commensal bacteria to maintain intestinal mucosal immune homeostasis (72, 73). Propionate and butyrate regulate B-cell class-switch recombination, somatic hypermutation, and plasma cell differentiation by inhibiting HDACs at physiological concentrations, inducing hyperacetylation of related gene loci to support B-cell differentiation into plasma cells and IgA production (74). In addition, pentanoate can also block the differentiation of CD4+ T cells into pathogenic Th17 cells, reduce IL-17A secretion, induce regulatory B cells (Bregs), and inhibit their apoptosis, enhance lymphocyte glycolysis and IL-10 secretion by inhibiting HDACs, thereby exerting an immunosuppressive effect in colitis and multiple sclerosis models (75).

#### Innate immunity

Acetate promotes neutrophil recruitment to inflammatory sites and IL-1β release via FFAR2 to enhance innate immune responses. Meanwhile, in the intestine, acetate upregulates IL-1 receptor expression on innate lymphoid cell 3 (ILC3) through FFAR2 and stimulates IL-22 secretion upon IL-1β activation, which cooperates with neutrophils to strengthen anti-infective capacity and intestinal barrier repair (76). Propionate and butyrate inhibit the maturation and migration of DCs, induce the generation of tolerogenic DCs, promote the production of Tregs, and also suppress mast cell degranulation and inflammatory cytokine release (77). Butyrate exhibits the strongest HDACs inhibitory activity and exerts broad-spectrum regulation on innate immunity (77). It reprograms macrophage metabolism from glycolysis to oxidative phosphorylation (OXPHOS) and fatty acid oxidation (FAO), promotes M2 anti-inflammatory polarization, enhances LC3-mediated phagocytosis and antimicrobial activity, and inhibits the NF-κB inflammatory pathway (76). Butyrate also induces the expression of IDO1 and Aldh1A2 in DCs, suppresses the differentiation of naive T cells into IFN-γ-positive cells, and promotes the generation of FoxP3^+^ Tregs to maintain immune tolerance (78). In addition, butyrate modulates the phenotype and cytotoxic activity of NK cells and inhibits the activation of lung ILC2 to alleviate allergic inflammation (76). Pentanoate exerts anti-inflammatory effects through dual pathways of GPR41/43 signaling and HDACs inhibition, and suppresses the activation of mast cells and macrophages (77). It also inhibits IL-17A production and Th17 cell differentiation via metabolic-epigenetic crosstalk and induces the secretion of IL-10 by lymphocytes and Bregs. Furthermore, pentanoate shows no B-cell cytotoxicity as observed with butyrate (butyrate at 5 mM causes nearly 100% B-cell apoptosis) and markedly inhibits B-cell apoptosis while enhancing its anti-inflammatory function (75).

### Distant effects of SCFAs

#### Gut-liver axis

A large number of SCFAs are rapidly metabolized by colon cells in the intestine, and another part is absorbed in the hepatic vein and portal vein system. They maintain the homeostasis of the gut-liver axis through a two-way pathway of “intestinal barrier maintenance-liver direct protection” (79). On the intestinal end: SCFAs (especially butyrate) upregulate tight junction proteins, promote goblet cells to secrete MUC2 to thicken the mucus layer, and activate GPR43 to induce antimicrobial peptides (RegIIIγ). These actions reduce intestinal permeability and the entry of LPS into the liver, blocking the cause of liver damage from the source. On the hepatic end: SCFAs promote Treg differentiation and increase IL-10 release by inhibiting HDACs activity, and block the nuclear translocation of NF-κB to reduce pro-inflammatory factors, such as TNF-α and IL-6, thereby exerting anti-inflammatory effects. They reduce reactive oxygen species (ROS) damage, lowering the content of malondialdehyde (MDA, an oxidative stress marker) and the activity of myeloperoxidase (MPO, an indicator of neutrophil infiltration) in liver tissue to resist oxidation. Propionate activates the “acetylated histone 3/forkhead box protein O1 (FOXO-1)/heme oxygenase 1 (HO-1)” pathway to inhibit hepatocyte apoptosis and directly protect hepatocytes (80). However, under pathological conditions (such as hepatic ischemia-reperfusion injury, HIRI), gut microbiota imbalance leads to a decrease in SCFA-producing bacteria and downregulation of MCT1/SMCT1 expression. Insufficient SCFA production and transport exacerbate gut-liver axis disorder.

#### Gut-lung axis

**Fig 3:**
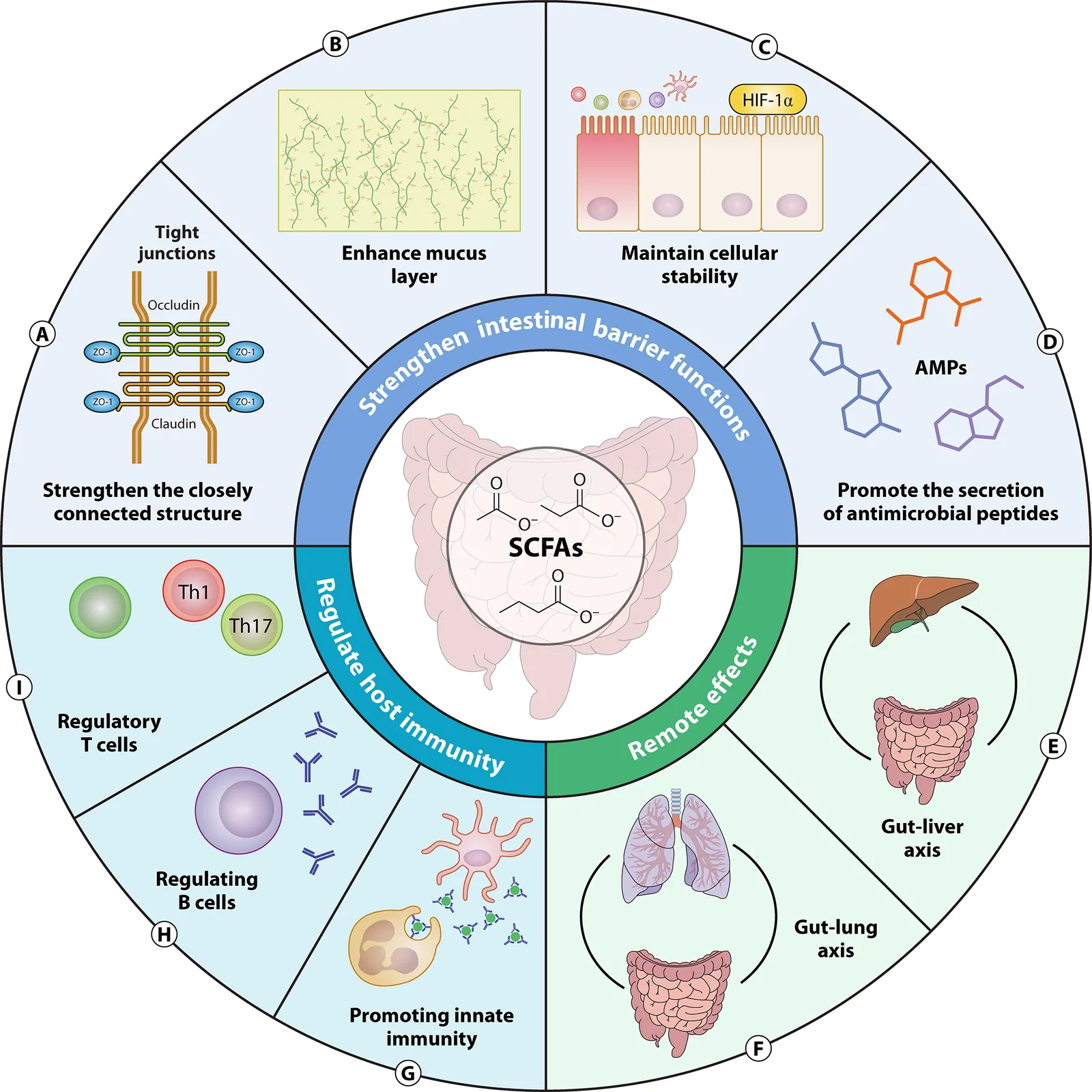
The regulatory effects of SCFAs on the intestinal barrier, host immunity, and distant organs. SCFAs enhance intestinal barrier functions: (**A**) SCFAs upregulate the expression of ZO-1 and Claudin-1 and downregulate Occludin-2 to strengthen tight junction structure. (**B**) SCFAs promote MUC2 expression, increasing mucus layer thickness, which physically prevents direct contact between gut microbiota and intestinal epithelium and provides a colonization environment for symbiotic bacteria, maintaining intestinal microecological balance. (**C**) SCFAs alleviate inflammatory responses and stabilize HIF-1α to maintain cellular homeostasis. (**D**) SCFAs promote the secretion of AMPs. Regulatory effects of SCFAs on organs outside the gut: (**E**) Gut-liver axis–SCFAs reach the liver via the portal vein circulation, participating in regulating inflammatory responses, antioxidation, and protecting hepatocyte function. (**F**) Gut-lung axis–SCFAs alleviate lung inflammatory damage through GPRs and HDACs inhibition, regulating bone marrow hematopoiesis to strengthen pulmonary immune defense. SCFAs regulate host immunity: (**G**) they act on dendritic cells, neutrophils, and other immune cells to enhance innate immunity and inhibit excessive inflammation; (**H**) modulate B cell differentiation, IgA/IgG production, and regulatory B cell induction; (**I**) and promote Treg differentiation and balance Th1/Th17 polarization.

SCFAs can enter the systemic circulation through passive diffusion or active transport, and then reach the lungs to exert systemic effects (81). Similar to the gut-liver axis, they maintain the homeostasis of the gut-lung axis through two-way protection of the intestinal and pulmonary ends. The mechanism at the intestinal end is similar to that of the gut-liver axis. On the pulmonary end: SCFAs regulate gut-lung axis-related immune responses through multiple targets. First, activating GPRs on the surface of pulmonary immune cells. For example, GPR109a promotes the secretion of anti-inflammatory factor IL-10 by activating Tregs (82). Second, by inhibiting HDACs, they inhibit the activity of LPS-induced nitric oxide synthase (iNOS) and the activation of nuclear factor-κB (NF-κB) pathway, reducing excessive pulmonary inflammation (82). In addition, SCFAs can also promote the generation of DC precursor cells and macrophages with strong phagocytic capacity by regulating the hematopoietic process in the bone marrow. After migrating to the lungs, these cells can enhance the ability to clear pathogens, further strengthening pulmonary immune defense (83, 84). When gut microbiota imbalance leads to insufficient SCFA production, the homeostasis of the gut-lung axis is disrupted. The number of anti-inflammatory immune cells (Tregs, alternatively activated macrophages, AAMs) in the lungs decreases, increasing the risk of diseases, such as allergic airway inflammation (AAI) and influenza-related lung injury. When pulmonary infection occurs, it activates the systemic inflammatory response (increased IFN-γ and TNF-α), leading to the apoptosis of intestinal epithelial cells and damage to the intestinal barrier. At the same time, pulmonary inflammatory signals inhibit the proliferation of SCFA-producing bacteria in the intestine through the blood circulation, leading to the overgrowth of pathogenic bacteria and gut microbiota imbalance (83) (Fig. 3).

## THE ROLE OF SCFAs IN INFECTION TREATMENT

### Therapeutic potential of SCFAs in bacterial infections

#### SCFAs and *Staphylococcus aureus*

*Staphylococcus aureus* is a common colonizing bacterium in humans and one of the most important opportunistic bacterial pathogens. It causes severe morbidity and mortality worldwide (85). Studies have confirmed that SCFAs, as immunomodulatory metabolites, show potential in fighting *S. aureus* infections (86, 87).

In dairy cows, *S. aureus* is the most common infectious and opportunistic pathogen of dairy cow mastitis (88). SCFAs can directly inhibit the adhesion and invasion of *S. aureus*: they reduce bacterial attachment by downregulating the expression of adhesion molecules such as ICAM1 and VCAM1 in host cells (89, 90). For example, NaB can significantly reduce the mRNA levels of *S. aureus* adhesion-related gene *ClfB* and biofilm formation gene *SdrC* to inhibit its colonization (91). Meanwhile, SCFAs enter bacterial cells in a non-ionized form and dissociate to cause intracellular acidification, blocking the invasion process (92). Different SCFAs have different inhibition rates on *S. aureus* invasion into bovine mammary epithelial cells (bMECs), and 5 mM sodium acetate achieves an inhibition rate of 80% (93). SCFAs also enhance host defense: upregulating the gene expression of tracheal antimicrobial peptide (TAP) and bovine neutrophil β-defensin 5 (BNBD5) in bMECs to kill bacteria, activating the TLR2/p38 pathway via NaB to activate the antibacterial defense and promote NO production (94–96). Additionally, SCFAs inhibit inflammatory pathways (such as NF-κB and MAPKs pathways) to reduce the secretion of pro-inflammatory factors and enhance the expression and function of tight junction proteins to protect the blood-milk barrier (94, 97, 98). They can also regulate the gut-mammary axis, maintain microbiota balance, promote the secretion of mucus and defensins, and reduce bacterial translocation and overflow (99–101).

*S. aureus* not only threatens mammary health but also is a major pathogen of osteomyelitis. Osteomyelitis is a common infectious disease that may develop into osteonecrosis or bone degeneration (102). As the main pathogenic bacterium among Gram-positive bacteria, *S. aureus* accounts for more than 60% of osteomyelitis cases (103, 104). Studies have shown that butyrate can reduce bone loss by regulating the expression of m6A methyltransferase (METTL3) (105). Specifically, 10–20 μg/mL butyrate can effectively inhibit the infection-induced high expression of METTL3—whose levels are negatively correlated with the expression of osteogenic markers (such as RUNX2, OCN, and ALP)—thereby reversing the downregulation of osteogenic markers and restoring osteoblast function (105). However, when the concentration is ≥200 µg/mL, it significantly inhibits cell proliferation and aggravates cell damage after infection (105).

#### SCFAs and *Clostridioides difficile*

*Clostridioides difficile* is a Gram-positive, obligate anaerobic, spore-forming intestinal opportunistic pathogen. Spores enhance its aerobic survival and fecal-oral transmission risk (106). As a core metabolite of gut microbiota-fermented dietary fiber, SCFAs exert dual effects on *C. difficile*: inhibiting growth and promoting toxin production—an adaptive strategy confirmed by many *in vivo* and *in vitro* experiments (107).

##### Inhibitory effect of SCFAs on *C. difficile—in vivo* experiments

Hamster models show elderly hamsters with higher intestinal SCFAs have lower susceptibility to *C. difficile* infection (CDI) (108). Antibiotic treatment disrupts gut microbiota, reducing intestinal SCFAs—a marker of CDI susceptibility verified in humans and mice (109–112). *In vitro*, various SCFAs reduce *C. difficile* growth rate in a dose-dependent manner, with valerate showing obvious inhibitory effects on its vegetative cells (107, 110, 113, 114). Promoting effect of SCFAs on *C. difficile* toxins—*C. difficile* toxins TcdA and TcdB are regulated by transcription factors like TcdR (positive regulation), TcdC (negative regulation), CodY, CcpA, and Rex. SCFAs affect these factors via metabolic status (107). *In vitro*, adding SCFAs upregulates TcdA and TcdB expression. When cysteine and mixed amino acids in the medium are depleted, butyrate accumulates in *C. difficile* cells to enhance toxin synthesis (115). Animal experiments show high-fiber diets increase intestinal SCFAs in mice, raising toxin production per *C. d*ifficile cell short-term, but long-term fiber intake eliminates *C. difficile* via microbiota restoration, reducing total toxin levels (107, 110).

##### Different SCFAs have distinct anti-*C*. *difficile* mechanisms

Acetate binds to the receptor FFAR2 to form the “acetate-FFAR2 axis,” recruiting neutrophils to inflammatory sites and promoting IL-1β secretion. It also upregulates the expression of IL-1R in ILC3s to promote IL-22 production, preventing *C. difficile* from transferring to extra-intestinal tissues such as the liver and spleen, inducing antimicrobial peptide production, and activating the complement pathway (116, 117). Butyrate stabilizes HIF-1 to enhance barrier integrity, while activating GPRs to recruit neutrophils and induce the production of colonic antimicrobial peptides (reg3b, reg3g) (118). Valerate directly and specifically inhibits the growth of *C. difficile*. *In vitro*, valerate inhibits the proliferation of its vegetative cells without affecting symbiotic bacteria such as Bacteroidetes and Firmicutes (114). *In vivo*, oral administration of TB reduces the number of viable *C. difficile* in the feces of CDI mice by 95%. After fecal microbiota transplantation, the valerate level increases, which is directly related to the reduction of viable *C. difficile* (by 94%) and spores (by 86%) (116).

### SCFAs and *Salmonella enterica* serovar Typhimurium

SCFAs produced by gut microbial fermentation can effectively defend against intestinal colonization by *Salmonella enterica* serovar Typhimurium (*S. Typhimurium*) (119). Cellular and animal experiments confirmed that SCFAs directly bind to the lysine-rich region in the PYRIN domain (−KKFKLK−) of apoptosis-associated speck-like protein (ASC), promote the interaction between ASC and *NLRP* family proteins, and activate the inflammasome complex, thereby inducing macrophage pyroptosis to efficiently eliminate intracellular *S. Typhimurium*. Meanwhile, SCFAs promote IL-1β release and neutrophil recruitment to limit systemic bacterial dissemination, and this protective effect is independent of the classic SCFAs receptor GPR43 (120).

Cellular experiments showed that treatment with 10 mM propionate or butyrate significantly reduced intracellular bacterial load and increased the levels of lactate dehydrogenase (LDH, a pyroptosis marker), activated caspase-1, and IL-1β in *S. Typhimurium*-infected bone marrow-derived macrophages (BMDMs) after 8 hours (120). The protective effects were completely abolished after ASC knockout, but not affected by GPR43 knockout. In addition, the MCT inhibitor SR13800 (≥20 µM) attenuated the bacterial clearance and IL-1β induction effects of SCFAs (120). These results confirm that ASC is a key molecule for SCFAs to exert anti-*Salmonella* effects.

Animal experiments further demonstrated that SPF wild-type mice infected with *S. Typhimurium* had significantly higher survival rates than ASC-knockout mice. After antibiotic pretreatment (to clear endogenous SCFAs), there was no significant difference in survival rates between wild-type and ASC^−/−^ mice. Supplementation with 300 mM propionate or butyrate markedly improved the survival rate of wild-type mice but showed no effect on ASC^−/−^ mice (120). Moreover, supplementation with propionate, butyrate, or fermentable dietary fiber (partially hydrolyzed guar gum) reduced bacterial loads in the cecum, mesenteric lymph nodes, liver, and spleen of infected mice, and these effects were also dependent on ASC (120).

**Fig 4:**
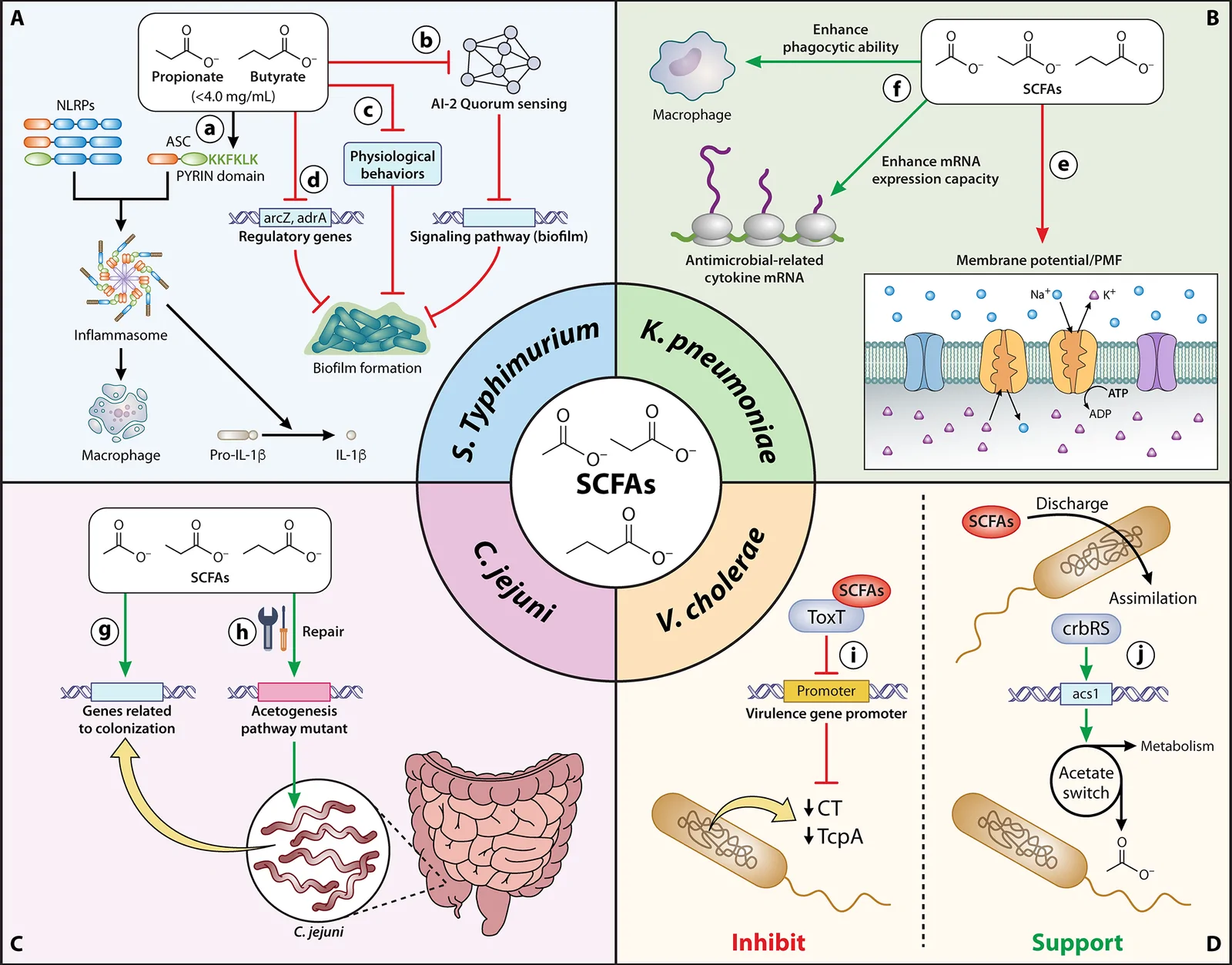
Interactions between SCFAs and bacteria. (**A**) SCFAs on *S. Typhimurium*: by (a) SCFAs bind to the lysine-rich region in the PYRIN domain of ASC, promote the interaction between ASC and NLRPs to activate the inflammasome complex, induce macrophage pyroptosis to eliminate intracellular bacteria, and facilitate IL-1β secretion to recruit neutrophils, thereby restricting systemic bacterial dissemination. SCFAs inhibit *S. Typhimurium* biofilm formation: propionate/butyrate (b) inhibit AI-2-mediated quorum sensing signaling pathways, (c) interfere with bacterial physiological behaviors, (d) downregulate the expression of key biofilm regulatory genes, such as *arcZ* and *adrA*. (**B**) SCFAs on *K. pneumoniae*: (e) disrupt membrane potential and PMF, blocking ATP synthesis and nutrient transport; (f) enhance host innate immunity, promote macrophage phagocytic function, upregulate mRNA expression of antimicrobial-related cytokines, and strengthen immune clearance at the infection site. (**C**) SCFAs on *C. jejuni*: (g) activate colonization-related genes, enhancing its epithelial colonization ability; (h) repair gene expression defects in acetogenesis pathway mutants. (**D**) SCFAs on *V. cholerae*: (i) inhibit virulence gene expression, interfere with virulence regulators, such as ToxT, suppress virulence gene promoter activity, downregulate expression of CT, TcpA, etc., reducing pathogenicity; (j) regulate metabolic homeostasis, participate in the “acetate switch” regulation, and affect acetate release and assimilation.

In addition, studies confirm that propionate and butyrate can inhibit the formation of *S. Typhimurium* SL1344 biofilm in LB broth or food models (113). At sub-inhibitory concentrations (SICs, <4.0 mg/mL), propionate and butyrate synergistically inhibit the formation of *S. Typhimurium* SL1344 biofilm through multiple pathways (121): they reduce the activity of AI-2 quorum sensing, blocking the signaling pathway for bacteria to form biofilms together, and downregulating the expression of biofilm key regulatory genes such as *arcZ* and *adrA* to reduce the synthesis of biofilm structural substances. Only butyrate can inhibit bacterial swimming motility in a dose-dependent manner, and both can reduce the adhesion ability of bacteria to the surface, thereby interfering with bacterial physiological behaviors. Furthermore, they reduce the metabolic activity of biofilms (butyrate is more effective), and scanning electron microscopy shows that the biofilm density decreases and gaps appear, damaging the structural integrity (121) (Fig. 4).

### SCFAs and other pathogenic bacteria

#### Klebsiella pneumoniae

SCFAs mainly exert growth inhibitory effects (not bactericidal effects) on *K. pneumoniae*, which depend on concentration, pH, oxygen environment, and alkyl chain length (122). SCFAs significantly inhibit bacterial growth only at concentrations of 50 mM or higher, with the strongest inhibitory effect observed at 100 mM, whereas no significant effect is detected at low concentrations (10 mM) (122). Furthermore, the inhibitory effect is further enhanced under anaerobic conditions and increases gradually with the elongation of the alkyl chain length (C2 < C3 < C4 < C6) (122). In addition, the inhibitory effects of SCFAs are much stronger in the cecum/ascending colon at pH 5.75 than in the neutral environment at pH 7.0 (8). This is because at pH 5.75, SCFAs penetrate the bacterial membrane in a protonated form and dissociate intracellularly to disrupt membrane potential and proton motive force (PMF), thereby blocking ATP synthesis and nutrient transport (122). Although intestinal symbiotic bacteria (such as *Bacteroides fragilis*) cannot directly inhibit this bacterium, their supernatant rich in acetate can indirectly inhibit the growth of the strain under anaerobic/pH 5.75 conditions (122). SCFAs can also enhance the phagocytic ability of alveolar macrophages, upregulate the mRNA expression of antibacterial-related cytokines such as IL-6 and TNF-α (by 2–3 times), and recruit immune cells such as neutrophils to synergistically clear bacteria. At the same time, they reduce the pulmonary pathological score to reduce excessive inflammatory damage (123).

#### Campylobacter jejuni

SCFAs play a supporting role in *C. jejuni* and are key signals for it to recognize the intestinal niche. SCFAs can directly activate the colonization-related genes of *C. jejuni* (such as *ggt* and peb1c, which encode amino acid transport/metabolism proteins) and repair the gene expression defects of acetogenesis pathway (acetate-producing pathway, with pyruvate as the starting substrate) mutants (124). In addition, SCFAs have a high content in the distal intestine (such as the cecum), which can guide *C. jejuni* to preferentially colonize the distal intestine, and can antagonize the inhibitory effect of high-concentration lactic acid in the upper intestine on colonization genes (125).

#### Vibrio cholerae

SCFAs exert dual regulatory effects on *V. cholerae*, and different types of SCFAs affect bacterial virulence and colonization through distinct mechanisms (125). On the one hand, acetate enhances the virulence and promotes the colonization of *V. cholerae. V. cholerae* can switch its metabolic mode from “acetate excretion” to “acetate assimilation” via the acetate switch mechanism regulated by the crbRS genes and mediated by the acs1 gene (126). The assimilation of acetate provides a growth advantage to *V. cholerae*, especially at the early stage of infection when bacterial abundance is low. For example, in a Drosophila model, knockout of the crbS or acs1 gene significantly reduces mortality caused by *V. cholerae*. Meanwhile, acetate metabolism disrupts the host insulin signaling pathway, induces intestinal steatosis, and ultimately exacerbates host damage and increases mortality (125). On the other hand, butyrate significantly inhibits the virulence of *V. cholerae* (127). Butyrate can directly bind to ToxT, the core virulence regulator of *V. cholerae* (128, 129), suppress its activation of downstream virulence genes, thereby reducing the expression of cholera toxin (CT) and toxin-coregulated pilus (TcpA), and weakening bacterial virulence, adhesion, and invasion ability (127) (Fig. 4).

### Therapeutic potential of SCFAs in fungal infections

#### SCFAs and *Candida albicans*

The colonization rate of *Candida albicans* in the human gastrointestinal tract is about 60%–80% (130, 131). SCFAs are key targets in regulating the interaction between *C. albicans* and the host, providing directions for infection treatment. Although *C. albicans* can metabolize SCFAs, multiple studies show that in the high millimolar concentration environment of the human and mouse intestines, acetate, butyrate, and propionate can inhibit the growth of *C. albicans* (132–137).

SCFAs weaken the pathogenicity of *C. albicans* through multiple mechanisms: first, they can directly inhibit fungal growth. Under experimental laboratory conditions, butyrate and propionate have a stronger inhibitory effect on the growth of *C. albicans* than acetate (132, 138). Second, it inhibits its transformation from the yeast form to the invasive hyphal form (this transformation is an important pathogenic feature) (139), specifically by regulating hypha-specific gene expression through altering chromatin structure (140), and induces its metabolism to shift from a “fermentative” type to a “respiratory” type (141–143), with factors such as strains, carbon sources, and pH affecting the inhibitory effect. Third, it alters the structure of its cell wall, mainly reflected in changes in β-glucan and chitin (139, 144). For example, butyrate reduces chitin synthesis (139); butyrate and acetate promote the exposure of β-glucan on the cell surface (butyrate depends on the Gpr1 pathway); propionate masks β-glucan (144).

In addition, SCFAs can indirectly control fungi by regulating host immunity. For example, they enhance the phagocytic and bactericidal abilities of macrophages (such as increasing NO production and slowing the growth of phagocytosed yeasts) and inhibit the phagocytic function of neutrophils (137). They regulate cytokine secretion: reducing IL-1β to alleviate excessive inflammation and promoting IL-17 and IL-22 to enhance anti-fungal immunity. These effects together maintain the balance of intestinal microecology and limit the excessive proliferation and invasion of *C. albicans*.

However, up to now, there is limited evidence on the effects of SCFAs on other fungi, and further studies are needed to confirm.

### Therapeutic potential of SCFAs in viral infections

#### SCFAs and severe acute respiratory syndrome coronavirus 2

Coronavirus disease 2019 (COVID-19) caused by acute respiratory syndrome coronavirus 2 (SARS-CoV-2) mainly attacks the respiratory system, but it can also involve the gastrointestinal tract (145), and it can also lead to changes in the gut microbiota—a decrease in microbial diversity, an increase in pathogenic bacteria, such as *Clostridium*, and a reduction in beneficial bacteria that produce SCFAs, such as *Prevotella* (146), with the number of *Prevotella* being inversely related to the severity of the disease (147). As the main metabolic product of gut microbiota, SCFAs interact with SARS-CoV-2 in multiple ways, and the virus also indirectly affects the disease course through the gut microbiota.

SCFAs can reduce the possibility of viral infection by reducing the expression of key receptors for SARS-CoV-2 to invade cells. Butyrate can significantly reduce the expression of virus infection-related genes, such as Tmprss2 and Ace2, and increase the expression of antiviral-related toll-like receptors (TLRs) (22). In terms of immune regulation, SCFAs can alleviate the excessive inflammation caused by SARS-CoV-2 infection: butyrate inhibits HDACs to promote the transformation of macrophages to the anti-inflammatory M2 phenotype, reducing the release of pro-inflammatory cytokines and alleviating the “cytokine storm” that aggravates severe COVID-19 (50). At the same time, it promotes Treg differentiation and the secretion of anti-inflammatory cytokine IL-10, maintaining immune stability and alleviating inflammatory damage to the lungs and systemic tissues (148, 149).

**Fig 5:**
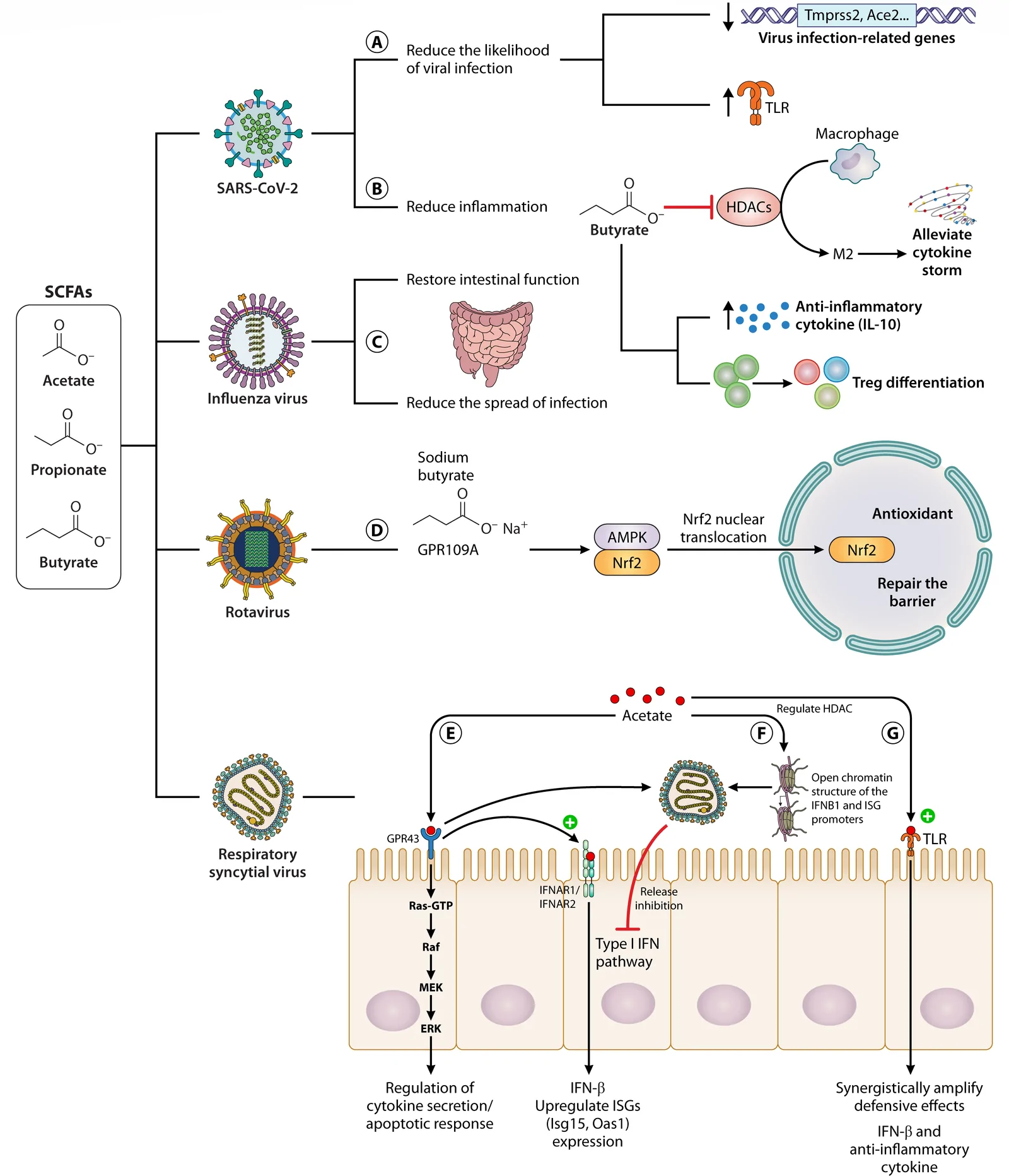
Interactions between SCFAs and viruses. SCFAs against SARS-CoV-2: (**A**) butyrate significantly reduces the expression of virus infection-related genes such as Tmprss2 and Ace2, while enhancing the expression of antiviral TLRs. (**B**) Butyrate, by inhibiting HDACs, promotes the polarization of macrophages towards the anti-inflammatory M2 phenotype, reducing the release of pro-inflammatory cytokines and alleviating the “cytokine storm” that contributes to severe disease; it also promotes Treg differentiation and IL-10 secretion, thereby mitigating inflammation in the lungs and systemic tissues. SCFAs against influenza virus (**C**): restore gut function and limit infection spread. SCFAs against rotavirus (**D**): sodium butyrate promotes Nrf2 nuclear translocation through the GPR109a-AMPK-Nrf2 pathway, thereby achieving antioxidant effects and repairing the gut barrier. SCFAs against RSV: acetate binds to GPR43 (**E**) and activates the Ras-ERK pathway to regulate cytokine secretion and apoptotic responses. It synergizes with IFNAR to promote IFN-β production and upregulate ISGs, while alleviating RSV-mediated inhibition of the type I interferon pathway. In addition, (**F**) acetate remodels the chromatin structure of the IFNB1 and ISG promoters by regulating HDAC activity to further relieve the suppression of the type I interferon pathway by RSV. (**G**) Acetate also synergizes with TLR signaling to amplify antiviral defense and promote the secretion of IFN-β and anti-inflammatory cytokines.

In addition, SARS-CoV-2 infection indirectly affects disease development through the gut microbiota: infection causes gut microbiota imbalance, reducing beneficial bacteria producing SCFAs and increasing opportunistic pathogens, leading to decreased SCFAs synthesis ability (146). The level of SCFAs in the feces of severe COVID-19 patients is significantly reduced, and it is negatively correlated with the severity of the disease, suggesting that SCFA deficiency may aggravate the disease (150). In addition, excessively low SCFA levels may affect pulmonary immune function through the “gut-lung axis” and increase the risk of pulmonary inflammation. Supplementing SCFAs or regulating microbiota may indirectly improve patient prognosis by restoring intestinal barrier function and reducing bacterial translocation. However, the number of subjects tested in existing studies is too small, so reliable conclusions cannot be drawn yet (Fig. 5).

#### SCFAs and influenza virus

Studies have shown that influenza virus infection can cause gut microbiota imbalance, thereby reducing SCFA production and promoting secondary pulmonary infections (151). Studies also confirm that influenza A virus (IAV) infection disrupts the gut microbiota structure of mice, leading to a significant decrease in SCFAs levels produced by gut microbiota fermentation. This decrease in SCFAs is closely related to the damage of intestinal barrier function, which is manifested by increased intestinal permeability, translocation of bacterial components to the liver causing inflammation, and increased susceptibility to secondary intestinal bacterial infections (such as *S. Typhimurium* infection).

Studies find that infecting mice with a sublethal dose of IAV leads to a series of intestinal inflammation and functional abnormalities, and the infection reaches a peak at 7 days. Compared with uninfected mice, 7 days after infection, IAV-infected mice show significantly shortened colon length (a marker of intestinal inflammation), increased intestinal NF-κB (indicating that the intestine is in a stressed and inflammatory state), and decreased blood citrulline concentration (a marker reflecting the function and metabolic activity of intestinal epithelial cells) (152). Supplementing SCFAs can reduce intestinal permeability, decrease the expression of inflammatory genes in the liver, alleviate colon shortening, and reduce the systemic spread of *S. Typhimurium*, and improve the survival rate of mice with dual infections. However, it should be noted that SCFAs cannot reverse the intestinal disorders during influenza virus infection (Fig. 5).

#### SCFAs and rotavirus

Studies confirm that NaB has a protective effect on intestinal epithelial barrier damage induced by rotavirus (RV). In experiments using porcine jejunal epithelial cells as a model, NaB at adaptive concentrations (2 mM, 4 mM) binds to the GPR109a receptor to activate the AMPK-Nrf2 signaling pathway, promoting the nuclear translocation of Nrf2. This upregulates the expression of antioxidant genes (such as SOD, CAT) and tight junction-related genes (such as occludin, claudin-1), ultimately achieving dual effects of antioxidation and barrier repair (153) (Fig. 5).

#### SCFAs and respiratory syncytial virus

A high-fiber diet can enrich acetate-producing bacteria (such as bacteria of the family *Lachnospiraceae*) and increase acetate levels in the body (154). Acetate specifically binds to the GPR43 receptor on the surface of lung epithelial cells and immune cells, triggering downstream signals: on the one hand, it activates the Ras-ERK pathway to regulate cytokine secretion and apoptotic responses; on the other hand, it synergizes with the type I interferon receptor (IFNAR) to enhance the production and signal transduction of IFN-β, and upregulates the expression of interferon-stimulated genes (ISGs), such as *Isg15* (encoding ISG15 protein) and *Oas1* (encoding oligoadenylate synthetase OAS) (154, 155). Among them, OAS can synthesize 2′−5′-oligoadenylate (2−5A), which activates RNase L to degrade RSV RNA and directly inhibit virus replication (156, 157). Respiratory syncytial virus (RSV) inhibits the host type I interferon pathway through its non-structural proteins (NS proteins). Acetate can relieve this inhibition in two ways: it activates and remodels cell metabolism through GPR43 (acetate can be converted into acetyl-CoA under the action of acetyl-CoA synthetase 2 [158]), providing energy for immune signaling pathways. It regulates histone acetylation, opening the chromatin structure of the promoters of *IFNB1* and ISGs, enhancing their transcriptional activity to offset the inhibitory effect of RSV NS proteins (30, 159). In addition, acetate can synergize with TLRs signals to amplify the defense effect, promoting the secretion of IFN-β and anti-inflammatory cytokines to further inhibit virus spread (30) (Fig. 5).

Collectively, corresponding characteristics and biological functions of SCFAs are summarized in Table 1.

**TABLE 1:** SCFAs: receptor affinity, epigenetic capacity, core functions, and anti-infective effects

SCFA	Receptor affinity	Epigenetic capacity	Core functions	Anti-infective effects
Acetate	FFAR2＞FFAR3 Olfr78(+) GPR109a(-)	Weak	Mediates distant effects Promotes IgA and mucosal immunity Recruits neutrophils, and induces IL- 22 Inhibits T-cell migration and activation	Inhibits: *Staphylococcus aureus, Clostridioides difficile, Candida albicans,* RSV, influenza virus Promotes: *Campylobacter jejuni, Vibrio cholerae*
Propionate	FFAR2≈FFAR3 Olfr78(+) GPR109a(-)	Moderate	Activates inflammasome Promotes antimicrobial peptide secretion	Inhibits: *Salmonella Typhimurium, Klebsiella pneumoniae, Staphylococcus aureus, Clostridioides difficile, Candida albicans,* SARS-CoV-2
Butyrate	FFAR3＞FFAR2 GPR109a(++) Olfr78(-)	Strongest	Energy supply Strengthens intestinal barrier Regulates immune cells Induces Treg/M2 polarization Stabilizes HIF-1α	Inhibits: *Staphylococcus aureus, Clostridioides difficile, Vibrio cholerae, Salmonella Typhimurium, Candida albicans*, SARS-CoV-2, Rotavirus
Valerate	Low affinity to GPRs	Strong	Anti-inflammation Induces Bregs Suppresses Th17 Low B-cell cytotoxicity	Inhibits: *Clostridioides difficile*

## TARGETED SCFA-BASED INFECTION TREATMENT STRATEGIES AND APPLICATION PROSPECTS

### SCFA intervention based on microbiota regulation

#### High-fiber diet/SCFA precursors promote the proliferation of SCFA-producing bacteria to enhance infection defense

Diet can selectively regulate the gut microbiota, promote the proliferation of SCFA-producing bacteria, thereby increasing intestinal SCFA levels and enhancing the body’s infection defense ability.

Dietary fiber is the core substrate for gut microbiota fermentation and can selectively enrich SCFA-producing bacteria. Research has demonstrated that consumption of a high-fiber diet results in the enrichment of SCFA-producing bacteria (160). Based on existing research data, dietary fiber intervention is expected to become an auxiliary strategy for infection treatment. Supplementing resistant starch can increase butyrate levels and enhance the intestinal resistance to bacterial infections. Inulin can enrich bacteria such as Bifidobacterium and *Roseburia* to increase SCFA concentrations, assisting in alleviating inflammation caused by intestinal infections (161). In addition, selecting dietary fiber for specific infection types (such as arabinoxylan to assist in regulating intestinal immunity [162]) may achieve more precise anti-infective regulation.

Diets rich in omega-3 (such as α-linolenic acid, eicosapentaenoic acid, docosahexaenoic acid) are also beneficial for microbiota regulation. In a randomized trial, supplementing omega-3 polyunsaturated fatty acids (PUFA) for 2 months increased the abundance of *Roseburia* and *Lactobacillus* in the gut of healthy people (39). Studies also show that after consuming an omega-3-rich diet for two weeks, the number of butyrate-producing bacteria increases significantly (163).

Studies confirm that dairy products fermented by beneficial bacteria or added with beneficial bacteria can increase SCFA-producing bacteria. For example, dairy products fermented by *Bifidobacterium animalis* subsp. *lactis* can increase butyrate-producing bacteria and cecal SCFAs in mouse models, and alleviate inflammation (164).

#### Probiotics/fecal microbiota transplantation: restore microbiota balance to increase endogenous SCFA levels

Fecal microbiota transplantation (FMT) aims to replace the imbalanced microbiota with healthy microbiota (165). It is a process of administering feces from healthy people containing thousands of bacterial groups to patients. Its core goal is to restore the healthy gut microbiota structure and gut microecological balance to exert therapeutic effects. FMT can indirectly increase intestinal SCFA levels by restoring SCFA-producing microbiota, and the two may have a synergistic effect in regulating the microbiota-immune axis.

### Exogenous SCFA supplementation therapy

#### Direct administration of SCFA preparations

SCFAs can be administered orally or rectally via enemas or suppositories to raise local concentrations (166, 167). Among these, rectal administration effectively avoids the hepatic first-pass effect and is more suitable for intestinal barrier repair in local intestinal infections and active ulcerative colitis (UC) (52).

Preclinical studies have shown that NaB alleviates LPS-induced intestinal injury (168), SCFA mixtures activate the NLRP6 inflammasome to improve sepsis-induced gut–brain axis damage (169), SCFA enemas inhibit *Shigella* infection (170), colonic infusion reverses intestinal mucosal atrophy (171), and rectal perfusion promotes epithelial proliferation (172). Clinical applications have confirmed that SCFA enemas repair mucosal injury in distal UC (173), dietary fiber supplementation corrects dysbiosis-related diarrhea in ICU patients (174), and oral sodium propionate reduces inflammation and oxidative stress in patients with chronic kidney disease (175), with favorable overall safety. In addition, two ongoing clinical trials (NCT04520594, NCT04522271) are also investigating resistant starch, a SCFA precursor, as an adjuvant therapy for pediatric inflammatory bowel disease to maintain disease remission by increasing endogenous SCFA levels (176). Preclinical and clinical evidences of SCFAs are summarized in Table 2.

**TABLE 2:** Preclinical and clinical studies on SCFAs

Type	Study model	Condition	Intervention	Effects	Reference
Preclinical	5-week-old SPF male SD rats	LPS-induced intestinal injury and microbial dysbiosis	NaB 4 g/kg by gavage for 30 days; LPS 100 μg/kg i.p. on day 31	Attenuated LPS-induced intestinal injury; downregulated TLR4/NF-κB pathway; reduced TNF-α, IL-6; repaired mucosal and mitochondrial damage; reversed gut dysbiosis	Dou et al. (139)
Preclinical	6–8-week-old male C57BL/6 mice	Sepsis-induced gut dysbiosis and hippocampal neuroinflammation	SCFA mixture (acetate: propionate: butyrate = 3:1:1) administered via drinking water	Activated colonic NLRP6 inflammasome; restored gut barrier and microbiota; reduced hippocampal neuroinflammation; improved cognitive function	Zhao et al. (140)
Preclinical	Adult rabbit models	*Shigella flexneri* 2a-induced acute colitis	SCFA enema (acetate 60 mM + propionate 30 mM + butyrate 40 mM), 10 mL per 6 h	Improved clinical symptoms; alleviated colonic mucosal injury; inhibited Shigella growth; exerted anti-inflammatory, mucosal repair, and antibacterial effects	Rabbani et al. (141)
Preclinical	SD rats	Intestinal mucosal atrophy induced by fiber-free diet and cecectomy	Continuous intracolonic infusion for 7 days: SCFA mixture or butyrate (20/40 mM)	Reversed intestinal mucosal atrophy; promoted colonic mucosal growth and proliferation	Kripke et al. (142)
Preclinical	8-week-old male Wistar rats	Impaired intestinal epithelial renewal induced by fiber-free elemental diet	SCFA solution (acetate 150 mM + propionate 60 mM + butyrate 60 mM), rectal instillation 1 mL, three times daily for 5 days	Significantly increased crypt cell proliferation rate in jejunum, ileum, and distal colon; accelerated epithelial renewal and barrier repair	Ichikawa et al. (143)
Clinical	129 patients with distal ulcerative colitis	Intestinal mucosal inflammation and barrier dysfunction in distal ulcerative colitis	SCFA enema (acetate 60 mM + propionate 30 mM + butyrate 40 mM), 100 mL twice daily for 6 weeks	Relieved rectal bleeding and tenesmus; corrected abnormal crypt proliferation; repaired mucosal barrier	Cummings (144)
Clinical	13 ICU patients	Gut dysbiosis-induced diarrhea in critical illness	Soluble dietary fiber supplementation	Restored gut microbiota; increased fecal SCFA levels; improved diarrhea associated with dysbiosis	O'Keefe et al. (145)
Clinical	20 patients with chronic kidney disease	Inflammation and oxidative stress in chronic kidney disease	Oral sodium propionate	Reduced inflammation, oxidative stress, and uremic toxins; elevated IL-10; well-tolerated with no major adverse events	Magliocca et al. (146)

#### Combined therapy with SCFAs

Combined therapy with SCFAs has become an important research direction for the treatment of various diseases, achieving efficacy optimization through synergy with different intervention methods.

##### Combination with prebiotics

As fermentation substrates for intestinal flora, prebiotics (e.g., inulin, fructooligosaccharides, 2′-fucosyllactose) can promote the production of endogenous SCFAs, forming a synergistic model with the supplementation of exogenous SCFAs (177). For example, in the treatment of Parkinson’s disease (PD), oral administration of a compound preparation of propionic acid (1.200 mg/day) + butyric acid (2,400 mg/day) combined with the prebiotic 2′-fucosyllactose (3900 mg/day, delayed-release capsules), with intervention for 6 months on the basis of conventional PD treatment, can significantly improve patients’ motor function, reduce the daily levodopa equivalent dose, and simultaneously improve cognitive function (178). In active UC, the combination of SCFAs with inulin/FOS can significantly reduce the fecal calprotectin level of patients in only 7 days, decrease the endoscopic and histological inflammation scores of the rectal mucosa, and alleviate clinical symptoms such as intestinal bleeding and tenesmus (179).

##### Combination with antibiotics

**Fig 6:**
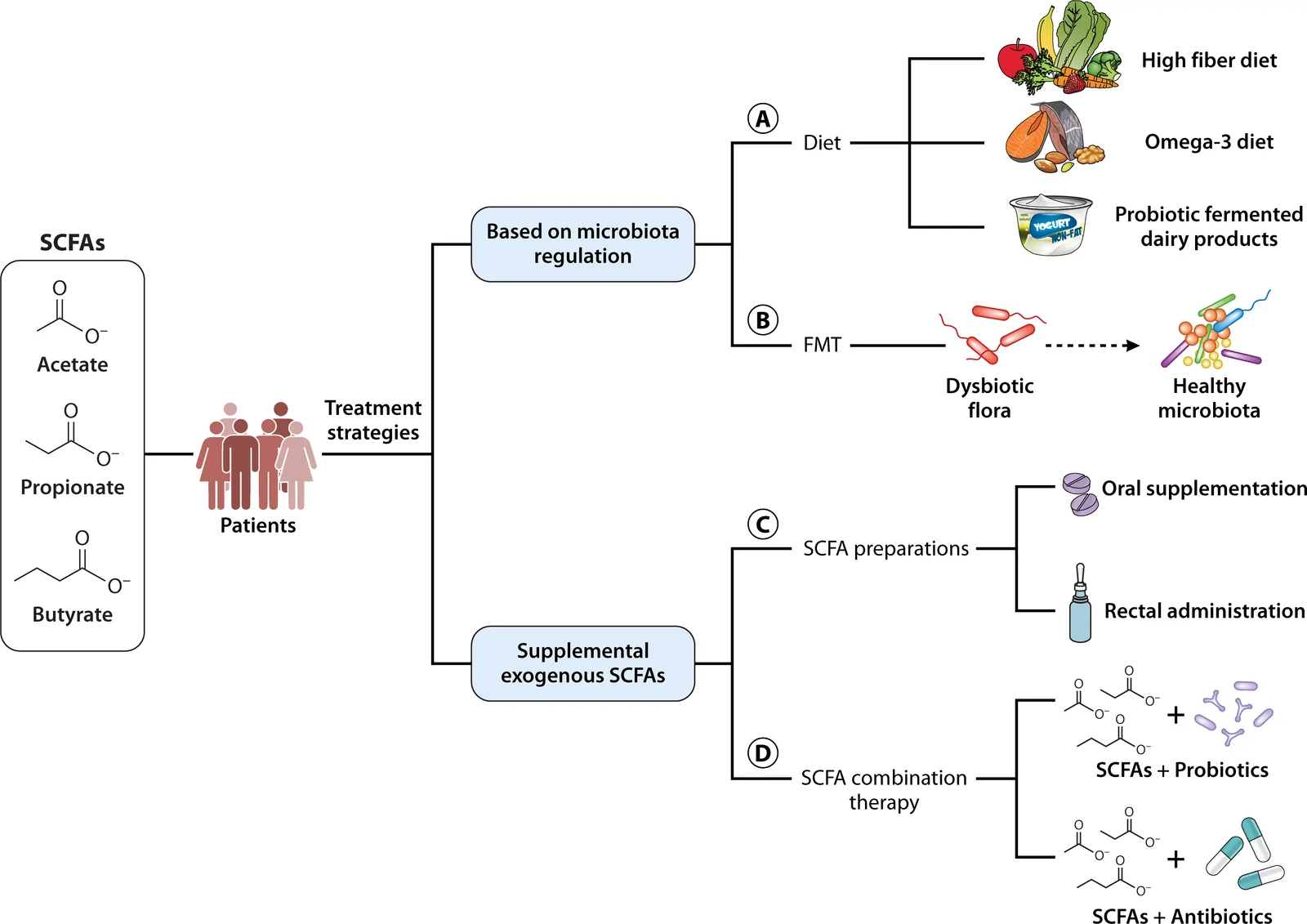
SCFA treatment strategies. Based on microbiota regulation: (**A**) Dietary intervention—high-fiber diets, omega-3 diets, and probiotic fermented dairy products can selectively enrich SCFA-producing bacteria and promote endogenous SCFA synthesis. (**B**) FMT: transplanting healthy microbiota to restore the abundance of SCFA-producing bacteria, indirectly increasing SCFA levels. Supplementation with exogenous SCFAs: (**C**) local concentrations of SCFAs are increased via oral or rectal administration; (**D**) combined therapy of “SCFAs + probiotics” and “SCFAs + antibiotics” to ensure efficacy, reduce antibiotic resistance, and maintain microbiota balance.

Although antibiotics can eliminate harmful pathogens, they also kill key SCFA-producing intestinal flora (e.g., amoxicillin-clavulanic acid can significantly reduce the abundance of *Roseburia* [180, 181]), disrupting the intestinal microecological balance. However, the combination of SCFAs with antibiotics can effectively alleviate this problem (182). In the intervention of antibiotic-related metabolic vascular injury, the use of vancomycin or antibiotic cocktails (including vancomycin, metronidazole, etc.) can lead to a decrease in the abundance of Bacteroidetes and insufficient acetate production, thereby exacerbating vascular calcification. Supplementation with acetate (e.g., 10 mM sodium acetate) can significantly reduce aortic calcium deposition, providing a combined adjuvant treatment idea of “antibiotics + acetate supplementation” for populations requiring long-term antibiotic use, which can effectively avoid antibiotic-induced metabolic vascular complications (183). In the treatment of gastrointestinal infection-related multidrug-resistant *Escherichia coli*, the combination of SCFAs (60–123 mM, simulating colonic physiological concentration) with β-lactam antibiotics/β-lactamase inhibitor combinations (e.g., cefoperazone/sulbactam, ceftazidime/avibactam), using SCFAs at 1/2 minimum inhibitory concentration (MIC) combined with antibiotics, can significantly reduce the MIC value of antibiotics, greatly improve the sensitivity rate of drug-resistant strains, and simultaneously inhibit the growth and pathogenic phenotype of *E. coli*, providing a new adjuvant treatment scheme for multidrug-resistant intestinal bacterial infections (184) (Fig. 6).

## DISCUSSION AND CONCLUSION

SCFAs, as key metabolites of the gut microbiota, exert anti-infective effects through metabolic and signal transduction functions, characterized by “predominant inhibition, minority anomalies.” They can inhibit most microorganisms, but promote the colonization of *C. jejuni*, and exert bidirectional regulation on *C. difficile* and *V. cholerae*.

Different types of SCFAs exert anti-infective effects through distinct pathways: as the most abundant SCFAs in the intestine, acetate mainly regulates immune cells and synergizes with multi-organ axes by activating the GPR43 receptor, exerting broad-spectrum anti-infective effects against bacteria (e.g., *C. difficile*) and viruses (e.g., RSV). Meanwhile, it exhibits a unique bidirectional property in supporting the virulence of *V. cholerae* via the “acetate switch.” Propionate can accurately clear intracellular bacteria (e.g., *S. Typhimurium*) and disrupt *S. Typhimurium* biofilms, while also assisting in regulating antiviral immunity and inhibiting the growth of Gram-negative bacteria, such as *K. pneumonia*. Butyrate is the core SCFAs for protecting the intestinal barrier: it regulates immunity by activating AMPK and inhibiting HDACs, and is effective against various pathogens but has the “butyrate paradox.” Valerate specifically inhibits *C. difficile*, kills its vegetative cells, reduces spore formation, and does not affect intestinal commensal bacteria.

However, current relevant studies are mostly based on animal experimental models, and the intestinal microenvironment of animals differs from that of humans. Therefore, large-scale clinical trials are still needed to verify the safety and efficacy of SCFAs in human applications. In addition, SCFAs exhibit dual effects in certain diseases (185), which suggests that clinical interventions must follow the principle of individualization.

Based on existing limitations, future research can advance in three directions. First, focus on clinical translation and verification: conduct multi-center, large-sample clinical studies, design gradient experiments of “dose-efficacy-safety” for specific infection types, and verify the clinical effects of combined regimens such as “SCFAs + probiotics” and “SCFAs + antibiotics” to provide evidence for their inclusion in anti-infective treatment guidelines. Second, deepen the exploration of the bidirectional effects of SCFAs and cell-specific regulatory factors, and improve the mechanism chain of SCFAs in anti-infection through signal transduction. Third, optimize intervention measures: develop new SCFA preparations to avoid the first-pass effect of the liver, design targeted drug delivery for extraintestinal infections, and combine individual microbiota characteristics to develop personalized combination regimens, thereby achieving precise anti-infective intervention.

In conclusion, given the complex interactions between SCFAs and the gut microbiota, as well as their regulatory role in infections, we believe that researching SCFAs is significant.
